# Decreased Peak Expiratory Flow Associated with Muscle Fiber-Type Switching in Spinal and Bulbar Muscular Atrophy

**DOI:** 10.1371/journal.pone.0168846

**Published:** 2016-12-22

**Authors:** Shinichiro Yamada, Atsushi Hashizume, Yasuhiro Hijikata, Tomonori Inagaki, Keisuke Suzuki, Naohide Kondo, Kaori Kawai, Seiya Noda, Hirotaka Nakanishi, Haruhiko Banno, Akihiro Hirakawa, Haruki Koike, Katherine Halievski, Cynthia L. Jordan, Masahisa Katsuno, Gen Sobue

**Affiliations:** 1 Department of Neurology, Nagoya University Graduate School of Medicine, Nagoya, Japan; 2 Innovation Center for Clinical Research, National Center for Geriatrics and Gerontology, Obu, Japan; 3 Institute for Advanced Research, Nagoya University, Nagoya, Japan; 4 Biostatistics Section, Center for Advanced Medicine and Clinical Research, Nagoya University Graduate School of Medicine, Nagoya, Japan; 5 Neuroscience Program, Michigan State University, East Lansing, Michigan, United States of America; 6 Research Division of Dementia and Neurodegenerative Disease, Nagoya University Graduate School of Medicine, Nagoya, Japan; Osaka University Graduate School of Medicine, JAPAN

## Abstract

The aim of this study was to characterize the respiratory function profile of subjects with spinal and bulbar muscular atrophy (SBMA), and to explore the underlying pathological mechanism by comparing the clinical and biochemical indices of this disease with those of amyotrophic lateral sclerosis (ALS). We enrolled male subjects with SBMA (*n* = 40) and ALS (*n* = 25) along with 15 healthy control subjects, and assessed their respiratory function, motor function, and muscle strength. Predicted values of peak expiratory flow (%PEF) and forced vital capacity were decreased in subjects with SBMA compared with controls. In SBMA, both values were strongly correlated with the trunk subscores of the motor function tests and showed deterioration relative to disease duration. Compared with activities of daily living (ADL)-matched ALS subjects, %PEF, tongue pressure, and grip power were substantially decreased in subjects with SBMA. Both immunofluorescence and RT-PCR demonstrated a selective decrease in the expression levels of the genes encoding the myosin heavy chains specific to fast-twitch fibers in SBMA subjects. The mRNA levels of peroxisome proliferator-activated receptor gamma coactivator 1-alpha and peroxisome proliferator-activated receptor delta were up-regulated in SBMA compared with ALS and controls. In conclusion, %PEF is a disease-specific respiratory marker for the severity and progression of SBMA. Explosive muscle strength, including %PEF, was selectively affected in subjects with SBMA and was associated with activation of the mitochondrial biogenesis-related molecular pathway in skeletal muscles.

## Introduction

Spinal and bulbar muscular atrophy (SBMA), or Kennedy’s disease, is a slowly progressive lower motor neuron and muscular disease characterized by bulbar and limb muscle weakness and elevated levels of serum creatine kinase [1–3]. SBMA is caused by the expansion of a CAG repeat within the first exon of the androgen receptor (*AR*) gene [4]. Muscular weakness generally appears between 30 and 60 years of age, and affected individuals typically require a wheelchair 15 to 20 years after the onset of symptoms [2]. Patients occasionally experience laryngospasm, a sudden sensation of dyspnea [5,6], and often develop dysphagia at advanced stages, eventually resulting in aspiration or choking. Pneumonia and/or respiratory failure may occur at advanced stages of the disease [7], indicating that the management of swallowing and respiratory function is indispensable for the long-term care of patients with SBMA. However, in contrast to amyotrophic lateral sclerosis (ALS), another motor neuron disease for which the clinical features of dyspnea have been well documented, respiratory impairment in SBMA has not been well characterized. For instance, forced vital capacity (FVC) is the most important respiratory marker in ALS and is critical for both the respiratory and nutritional management of patients [8,9], whereas such markers for SBMA management have yet to be identified. The aim of this study was to characterize respiratory function in subjects with SBMA both cross-sectionally and longitudinally, and to explore the potential underlying pathological mechanisms of SBMA by comparing the clinical and biochemical indices of this disease with those of ALS.

## Materials and Methods

### Standard protocol approvals, registration, and participant consent

This study conformed to the Ethics Guidelines for Human Genome/Gene Analysis Research and the Ethical Guidelines for Medical and Health Research Involving Human Subjects endorsed by the Japanese government. The Ethics Committee of Nagoya University Graduate School of Medicine approved this study, and all participants provided written informed consent prior to study participation.

### Study population

We studied 40 consecutive male subjects diagnosed with SBMA via genetic testing and 25 consecutive male subjects with a clinical diagnosis of definite to probable ALS based on the revised El Escorial Criteria [10]. We also evaluated 15 healthy, age-matched male subjects with no diagnosed neurological disorders. The inclusion criteria were as follows: (i) subjects were 30–80 years old at the time of informed consent, and (ii) subjects were able to stand upright for 6 min without assistance. The exclusion criteria were as follows: (i) severe complications, such as malignancy; (ii) other neurological complications; (iii) zero kg grip power in the dominant hand; or (iv) participation in any other clinical trial before providing informed consent. All subjects were Japanese males and were observed at Nagoya University Hospital between June 2013 and March 2016.

### Pulmonary function test

A pulmonary function test was performed for all participants using a spirometer (FUDAC-77; FUKUDA DENSHI, Tokyo, Japan), which calculated and recorded FVC, forced expiratory volume in 1 s (FEV_1.0_), the ratio of FEV_1.0_ to FVC, and peak expiratory flow (PEF). The predicted values of FVC and FEV_1.0_ were calculated using Baldwin’s equation [11] and Berglund’s equation [12], respectively. PEF is defined as the maximum expiratory flow per minute, which can be used to measure how fast a subject can exhale as well as to judge the strength of the expiratory muscles and the condition of the large airways. %PEF was calculated from regression equations for predicting PEF in the Japanese population. The subjects sat in a chair with a backrest and were instructed to inhale as deeply as possible, and then exhale through a mouthpiece as quickly as possible, with their noses occluded.

### Longitudinal analysis of pulmonary function tests

Pulmonary function was measured every 6 months. To clarify the chronological changes in respiratory function in subjects with SBMA, we analyzed the longitudinal data of subjects who were evaluated for 1 year or longer during the follow-up period.

### Motor function

We assessed disease severity in the subjects using the following functional parameters: the revised Amyotrophic Lateral Sclerosis Functional Rating Scale (ALSFRS-R), Spinal and Bulbar Muscular Atrophy Functional Rating Scale (SBMAFRS), modified quantitative myasthenia gravis (mQMG) score, tongue pressure, and grip power. SBMAFRS is a validated disease-specific functional scale for SBMA that demonstrates a high sensitivity for monitoring disease progression [13]. The validity of the motor functional measurements we used for SBMA is described in the S1 Methods.

### Immunohistochemistry of muscle biopsy specimens

Bicep muscle specimens for immunohistochemistry were obtained from male subjects with SBMA (*n* = 2; 44 and 56 years old) or with ALS (*n* = 2; 51 and 63 years) by open biopsy. The specimens were snap-frozen in isopentane, chilled in dry ice, and preserved at −80°C until analysis. Samples were cut on a cryostat using standard methods into 10-μm sections, as described previously [14]. Analysis of myosin heavy chain (MHC) expression was performed with primary antibodies against MHC type I (BAF-8; 1:50), MHC type IIa+IIx (SC-71; 1:600), and MHC type IIx (6H1; 1:50) (Developmental Studies Hybridoma Bank, University of Iowa) [15]. Immunoreactivity was detected using the following secondary antibodies: Alexa Fluor 647 IgG_2b_ (1:500), Alexa Fluor 488 IgG_1_ (1:500), and Alexa Fluor 555IgM (1:500) (Invitrogen, Carlsbad, CA, USA). Slides were visualized with an LSM710 laser-scanning confocal microscope (Carl Zeiss, Oberkochen, Germany).

### Quantitative RT-PCR

We analyzed the intramuscular mRNA expression levels of *MYH* genes in intercostal muscle specimens from 5 subjects with SBMA (mean ± SD age, 67.6 ± 10.6 years) and 5 subjects with ALS (68.4 ± 2.3 years). We also analyzed the mRNA levels of *MYH7* (encoding MHC type I), *MYH2* (encoding MHC type IIa), *MYH1* (encoding MHC type IIx), peroxisome proliferator-activated receptor alpha (*PGC-1α*), peroxisome proliferator-activated receptor alpha (*PPARα*), *PPARγ*, *PPARδ*, and *AMPK* in iliopsoas muscle specimens from 5 subjects with SBMA (67.6 ± 10.6 years), 6 subjects with ALS (64.8 ± 11.2 years), and 4 subjects with other diseases (70.8 ± 7.6 years), including progressive supranuclear palsy (*n* = 2), Guillain-Barré syndrome (*n* = 1), and Sjögren's syndrome (*n* = 1). There was no statistically significant difference in age at the time of examination between the SBMA subjects, ALS subjects, and disease controls. The detailed procedures are described in the S1 Methods.

### Genetic analysis

Genomic DNA was extracted from peripheral blood samples from subjects with SBMA using conventional techniques. PCR amplification of the *AR* CAG repeat was performed using a fluorescein-labeled forward primer (5′-TCCAGAATCTGTTCCAGAGCGTGC-3′) and an unlabeled reverse primer (5′-TGGCCTCGCTCAGGATGTCTTTAAG-3′). The detailed PCR conditions have been described previously [16].

### Statistical analysis

We used an unpaired *t*-test or Mann–Whitney U test to compare continuous variables between two groups, analysis of variance (ANOVA) with Tukey’s post-hoc test for multiple comparisons, and Pearson’s correlation coefficient for analyzing correlations among parameters. Analysis of covariance (ANCOVA) was performed to adjust the data for a covariate. We considered *p*-values less than 0.05 to be significant, and correlation coefficients (*r*) greater than 0.3 as strong. We fitted a marginal model using the generalized estimation equation (GEE) approach under an unstructured covariance matrix to clarify the population-averaged progression of the pulmonary function tests. Calculations were performed using the statistical software packages SPSS 23.0J (IBM Japan, Tokyo, Japan) and SAS 9.4 (SAS Institute Inc., NC, US).

## Results

### Clinical backgrounds and blood chemistry values of the subjects

The clinical backgrounds of the control subjects and the subjects with SBMA and ALS are presented in Table 1. The mean age at examination was higher in subjects with ALS than in those with SBMA, whereas the mean disease duration was shorter in the ALS subjects than in the SBMA subjects. The proportion of non-smokers, ex-smokers, and current smokers was equivalent in both groups. Serum concentrations of creatine kinase and testosterone were higher in the SBMA subjects. The characteristics of the SBMA subjects, such as age at examination, age at onset, and *AR* CAG repeat size, were similar to previously reported values [17–19].

**Table 1:** Clinical background of subjects.

	SBMA	ALS	Control	*p*-value^a^
	(*n* = 40)	(*n* = 25)	(*n* = 15)	
**Age at examination, years**	53.4 ± 10.4	65.2 ± 7.6	54.4 ± 7.7	<0.001
	(33–76)	(48–78)	(38–68)	
**Duration from onset, years**	8.7 ± 5.0	1.3 ± 0.9	NA	<0.001
	(0–17)	(0.5–4)		
**Smoking**				
**Non-smoker, %**	40.0	32.0	33.3	N.S.
**Ex-smoker, %**	45.0	52.0	26.7	N.S.
**Current smoker, %**	15.0	16.0	40.0	N.S.
**Blood chemistry values**				
** Creatine kinase, IU**	1057.8 ± 708.1	242.0 ± 276.3	119.7 ± 47.5	<0.001
	(202–3064)	(23–1383)	(43–204)	
**Testosterone, ng/mL**	7.8 ± 3.2	5.3 ± 1.6	6.8 ± 3.7	0.003
	(3.6–16.6)	(2.8–9.7)	(3.5–19.2)	
**CAG repeat size in *AR* gene**	47.2 ± 3.3	NA	NA	
	(42–54)			

SBMA, spinal and bulbar muscular atrophy; ALS, amyotrophic lateral sclerosis; NS, not significant; AR, androgen receptor; NA, not applicable. Data are shown as the mean ± SD.

^a^Difference between SBMA and ALS by ANOVA with Tukey’s post-hoc test.

### Respiratory function in SBMA

**Fig 1:**
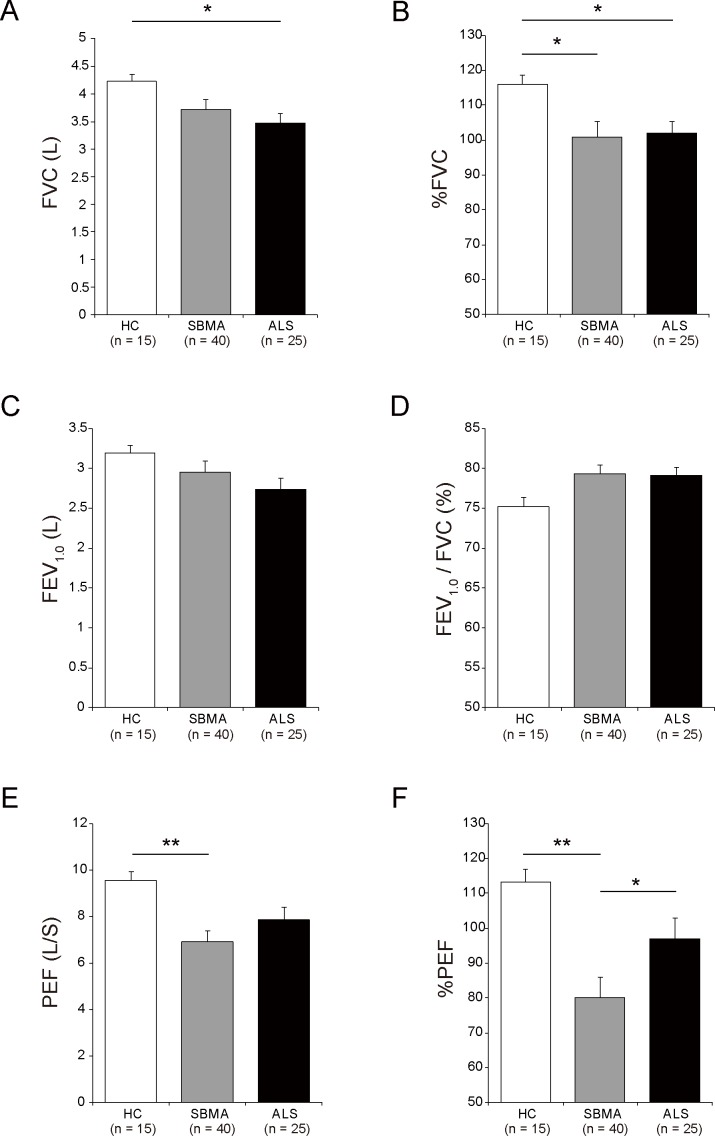
Respiratory function profile of subjects with SBMA. The actual and predicted values of forced vital capacity (FVC) (A, B), forced expiratory volume in 1 s (FEV_1.0_) (C), the ratio of FEV_1.0_ to FVC (D), and actual and predicted values of peak expiratory flow (PEF) (E, F) were compared among SBMA subjects (*n* = 40), ALS subjects (*n* = 25), or healthy controls (*n* = 15). Compared with the healthy controls, patients with SBMA exhibited decreased values for %FVC, PEF, and %PEF. The actual values of PEF were also lower in SBMA than in controls. When comparing SBMA and ALS subjects, %PEF was significantly decreased in SBMA, but no differences were detected for the other indices. ** *p* < 0.01. * *p* < 0.05. Data are presented as the mean ± SE. SBMA, spinal and bulbar muscular atrophy; ALS, amyotrophic lateral sclerosis; HC, healthy controls.

**Fig 2:**
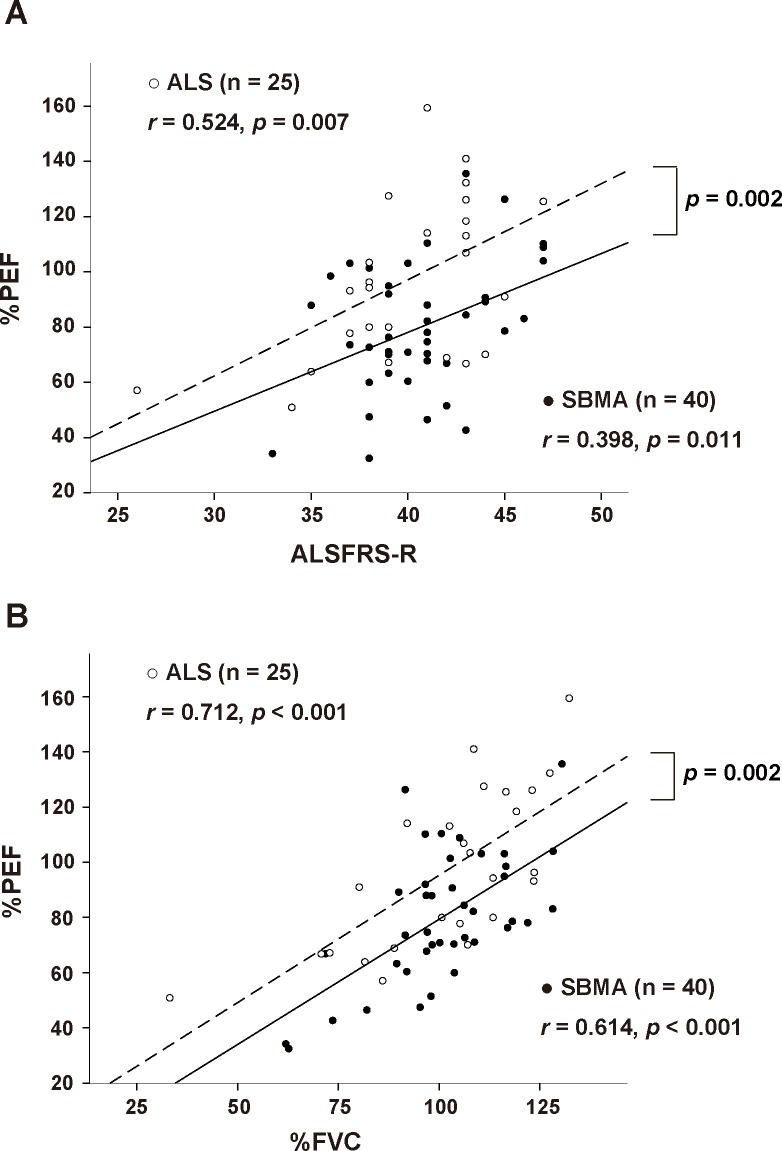
Relationships between %PEF and ALSFRS-R or %FVC in subjects with SBMA and ALS. Comparison of the relationships between %PEF and total score of ALSFRS-R (A) or %FVC (B) in SBMA and ALS. The difference in %PEF between SBMA and ALS was significant after adjustment for the ALSFRS-R and %FVC with ANCOVA. %PEF, predicted values of peak expiratory flow; ALSFRS-R, the revised Amyotrophic Lateral Sclerosis Functional Rating Scale; %FVC, predicted forced vital capacity.

Relative to healthy controls, the subjects with SBMA exhibited decreased values for %FVC and %PEF, but not for FEV_1.0_/FVC (Fig 1). The actual values for PEF were also lower in SBMA subjects than in control subjects. When comparing SBMA and ALS patients, the %PEF, an index of explosive muscle power, was significantly decreased in SBMA subjects, whereas other indices were comparable between the two groups. The difference in %PEF between SBMA and ALS subjects was significant after adjustment for the ALSFRS-R and %FVC with ANCOVA (*p =* 0.002 and 0.002, respectively) (Fig 2). These findings suggest that both %PEF and %FVC are decreased in SBMA patients, as observed in ALS, but the reduction of %PEF is specific to the subjects with SBMA.

### Relationship between respiratory function and motor function scores in SBMA

We next investigated the relationship between respiratory parameters and the total score and subscores on the motor functional scales SBMAFRS and ALSFRS in the SBMA subjects (Table 2). %PEF strongly correlated with total score as well as with the trunk and lower limb subscores of the SBMAFRS, particularly with the trunk subscore (S1 Fig). However, we observed no significant correlations between %PEF and bulbar, upper limb, or respiratory subscores. The lack of correlation between the respiratory domain of SBMAFRS and %PEF or %FVC appears to stem from the fact that most subjects were at relatively early stages of the disease and reported no overt dyspnea on the functional scales [13]. The total scores and subscores of SBMAFRS correlated more strongly with %PEF than %FVC. Similar relationships were also observed for ALSFRS-R (S2 Fig). These results indicate that %PEF is a sensitive biomarker of respiratory dysfunction, reflecting in particular the truncal function of subjects with SBMA.

**Table 2:** Correlations between motor function scores and respiratory indices in subjects with SBMA.

SBMA (*n* = 40)	%PEF	%FVC	FEV_1.0_ / FVC
**SBMAFRS**	*r* = 0.460 (*p =* 0.003)	*r* = 0.342 (*p* = 0.031)	*r* = 0.100 (*p* = 0.541)
**Bulbar**	*r* = 0.259 (*p* = 0.107)	*r* = 0.206 (*p* = 0.202)	*r* = −0.037 (*p* = 0.819)
**Upper Limb**	*r* = 0.007 (*p* = 0.964)	*r* = 0.017 (*p* = 0.918)	*r* = −0.142 (*p* = 0.383)
**Trunk**	*r* = 0.614 (*p* < 0.001)	*r* = 0.443 (*p* = 0.004)	*r* = 0.211 (*p* = 0.192)
**Lower Limb**	*r* = 0.379 (*p* = 0.003)	*r* = 0.248 (*p* = 0.123)	*r* = 0.260 (*p* = 0.105)
**Respiratory**	*r* = 0.063 (*p* = 0.699)	*r* = 0.076 (*p* = 0.642)	*r* = −0.003 (*p* = 0.983)
**ALSFRS-R**	*r* = 0.398 (*p* = 0.011)	*r* = 0.288 (*p* = 0.072)	*r* = 0.103 (*p* = 0.525)
**Bulbar**	*r* = 0.252 (*p* = 0.117)	*r* = 0.240 (*p* = 0.136)	*r* = 0.068 (*p* = 0.679)
**Upper Limb**	*r* = 0.002 (*p* = 0.992)	*r* = −0.078 (*p* = 0.632)	*r* = −0.156 (*p* = 0.337)
**Trunk**	*r* = 0.485 (*p* = 0.002)	*r* = 0.334 (*p* = 0.035)	*r* = 0.124 (*p* = 0.446)
**Lower Limb**	*r* = 0.331 (*p* = 0.037)	*r* = 0.271 (*p* = 0.091)	*r* = 0.199 (*p* = 0.219)
**Respiratory**	*r* = -0.032 (*p* = 0.699)	*r* = −0.057 (*p* = 0.725)	*r* = −0.008 (*p* = 0.960)

SBMA, spinal and bulbar muscular atrophy; %PEF, predicted values of peak expiratory flow; %FVC, predicted values of forced vital capacity; SBMAFRS, Spinal and Bulbar Muscular Atrophy Functional Rating Scale; ALSFRS-R, the revised Amyotrophic Lateral Sclerosis Functional Rating Scale.

### Longitudinal assessment of pulmonary function tests in SBMA

**Fig 3:**
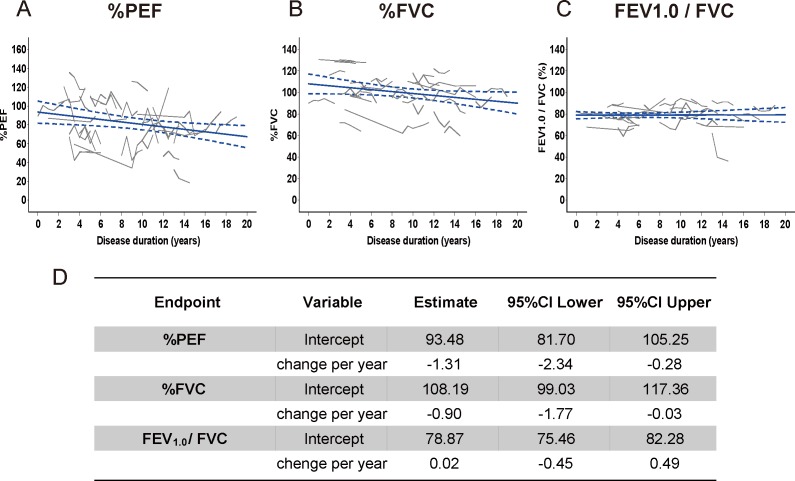
Longitudinal changes in respiratory function in SBMA. Longitudinal changes in %PEF (A), %VC (B), and FEV_1.0_/FVC (C) as a function of disease duration were analyzed. The solid lines indicate representative disease progression over disease duration calculated using a marginal model under an unstructured covariance matrix. The broken curvilinear line demonstrates the 95% confidence interval of these models. We calculated the estimated values at clinical onset (intercepts) and the change values per year (D). In an analysis using the marginal model, a generalized estimating equation (shown by the solid lines) identified that %PEF (A) and %FVC (B) demonstrated a slowly but with steady progression. PEF, peak expiratory flow; FVC, forced vital capacity; FEV_1.0_, forced expiratory volume in 1 s.

To examine whether respiratory parameters reflect disease progression, we prospectively analyzed longitudinal changes in pulmonary function tests in subjects with SBMA (Fig 3). Using a linear model, we assessed the data from 32 subjects with SBMA who were assessed for longitudinal changes in pulmonary function (Fig 3A–3C). Results revealed slow but steady deterioration for %PEF and %FVC relative to disease duration with the speed of decline being higher for %PEF. These results suggest that %PEF, together with %FVC, are biomarkers of respiratory function in subjects with SBMA and could be used to quantitatively assess disease progression.

### Fast versus slow motor function in SBMA and ALS

The decrease in %PEF in SBMA compared with ALS subjects led us to explore the possibility of a selective loss of fast muscle power in patients with this disease, considering that %PEF is an index of explosive muscle strength [20]. To test this hypothesis, we compared indices of fast and slow motor function that were not based on respiratory function between SBMA and ALS subjects. Both groups were matched for activities of daily living (ADLs) (Table 3). The total score and subscores (bulbar, upper limb, trunk, lower limb, and respiratory) on the ALSFRS-R in the SBMA subjects were equivalent to the scores of the ALS subjects. The mQMG scores, which are indices of muscle endurance, were also similar between the groups. Nevertheless, tongue pressure and grip power, both of which reflect explosive muscle strength, were significantly decreased in the SBMA subjects compared with the ALS subjects. These differences remained significant after adjustment for the ALSFRS-R with ANCOVA (data not shown). Taken together, our results suggest that explosive muscle power is preferentially affected in SBMA patients compared with ALS patients.

**Table 3:** Motor function of subjects.

	**SBMA**	**ALS**	**Control**	***p*-value**^b^
	(*n* = 40)	(*n* = 25)	(*n* = 15)	
**ALSFRS-R**				
**Total**	40.7 ± 3.3	39.9 ± 4.4	47.8 ± 0.6	N.S.
**Bulbar**	10.2 ± 1.1	10.0 ± 2.1	11.8 ± 0.6	N.S.
**Upper Limbs**	6.6 ± 0.9	6.7 ± 1.2	8.0 ± 0	N.S.
**Trunk**	5.8 ± 1.3	5.8 ± 2.0	8.0 ± 0	N.S.
**Lower Limbs**	6.2 ± 1.2	6.0 ± 1.2	8.0 ± 0	N.S.
**Respiratory**	11.9 ± 0.3	11.5 ± 1.1	12.0 ± 0	N.S.
**mQMG score**				
**Total**	5.48 ± 3.19	5.24 ± 3.31	ND	N.S.
**Head, lifted**	1.33 ± 0.86	1.32 ± 1.03	ND	N.S.
**Left arm outstretched**	1.33 ± 0.86	1.32 ± 1.03	ND	N.S.
**Right arm outstretched**	1.13 ± 0.82	1.28 ± 1.06	ND	N.S.
**Left leg outstretched**	0.85 ± 0.80	0.84 ± 1.07	ND	N.S.
**Right leg outstretched**	0.80 ± 0.72	0.68 ± 0.99	ND	N.S.
**Tongue pressure, kPa**	17.39 ± 6.64	25.89 ± 13.85	45.5 ± 7.31	0.003
	(5.3–32.0)	(0–47.0)	(34.4–55.1)	
**Grip power^a^, kg**	19.76 ± 5.91	26.58 ± 10.73	44.3 ± 6.23	0.002
	(7.7–31.9)	(8.4–49.3)	(29.2–52.0)	

SBMA, spinal and bulbar muscular atrophy; ALS, amyotrophic lateral sclerosis; ALSFRS-R, revised Amyotrophic Lateral Sclerosis Functional Rating Scale; mQMG, modified quantitative myasthenia gravis; NS, not significant; NA, not applicable; ND, not determined. ALSFRS-R normal value = 48. Data are shown as the mean ± SD.

^a^Maximum value of the dominant hand.

^b^Difference between SBMA and ALS by ANOVA with Tukey’s post-hoc test.

### Alteration of fast- and slow-twitch fiber composition in SBMA and ALS

**Fig 4:**
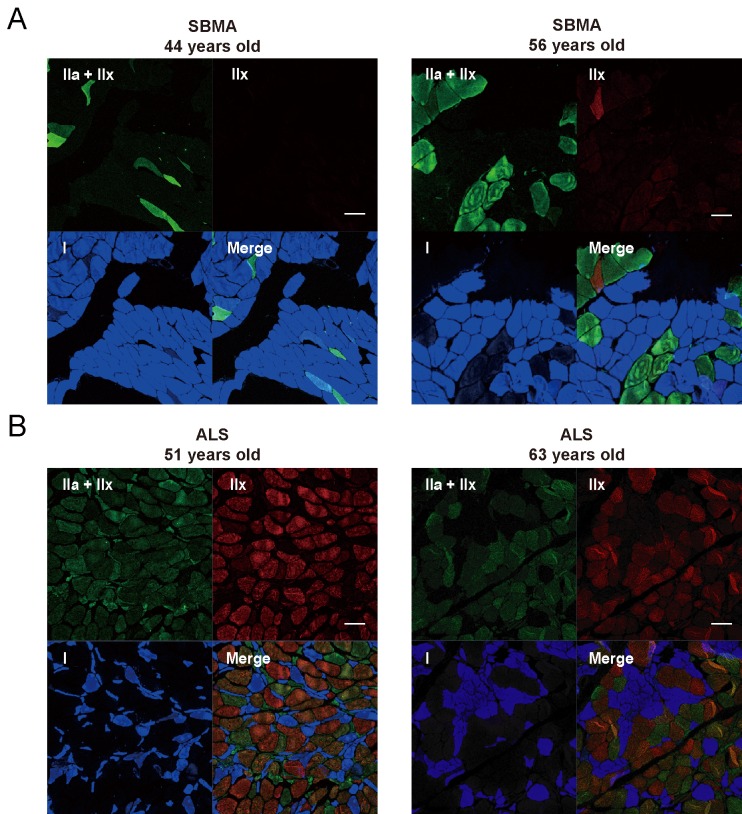
Disease-specific fiber type alterations in SBMA and ALS. (A, B) Representative images of anti-MHC immunostaining in the biceps muscles of subjects with SBMA (A, *n* = 2) and ALS (B, *n* = 2). Type I (blue), type IIa (strong green), and type IIx (strong red and intermediate green) fibers are shown in single- and merged-channel images of serial cross-sections of a human bicep incubated with an antibody cocktail (BAF-8, SC-71, and 6H1). Type IIx fibers were substantially decreased in SBMA compared with ALS, whereas type I fibers were atrophied in ALS compared with SBMA. Scale bar, 100 μm. SBMA, spinal and bulbar muscular atrophy; ALS, amyotrophic lateral sclerosis; MHC, myosin heavy chain.

Next, we hypothesized that the selective decline of fast muscle power in SBMA was attributable to an alteration in fast- and slow-twitch fiber composition. To examine this hypothesis, we analyzed MHC isoforms in biopsied skeletal muscle specimens using immunohistochemistry (Fig 4). Type IIx fibers, which generate explosive power, were substantially decreased in samples from subjects with SBMA compared with samples from subjects with ALS, whereas type I fibers, which are associated with endurance, were atrophied in samples from ALS subjects.

**Fig 5:**
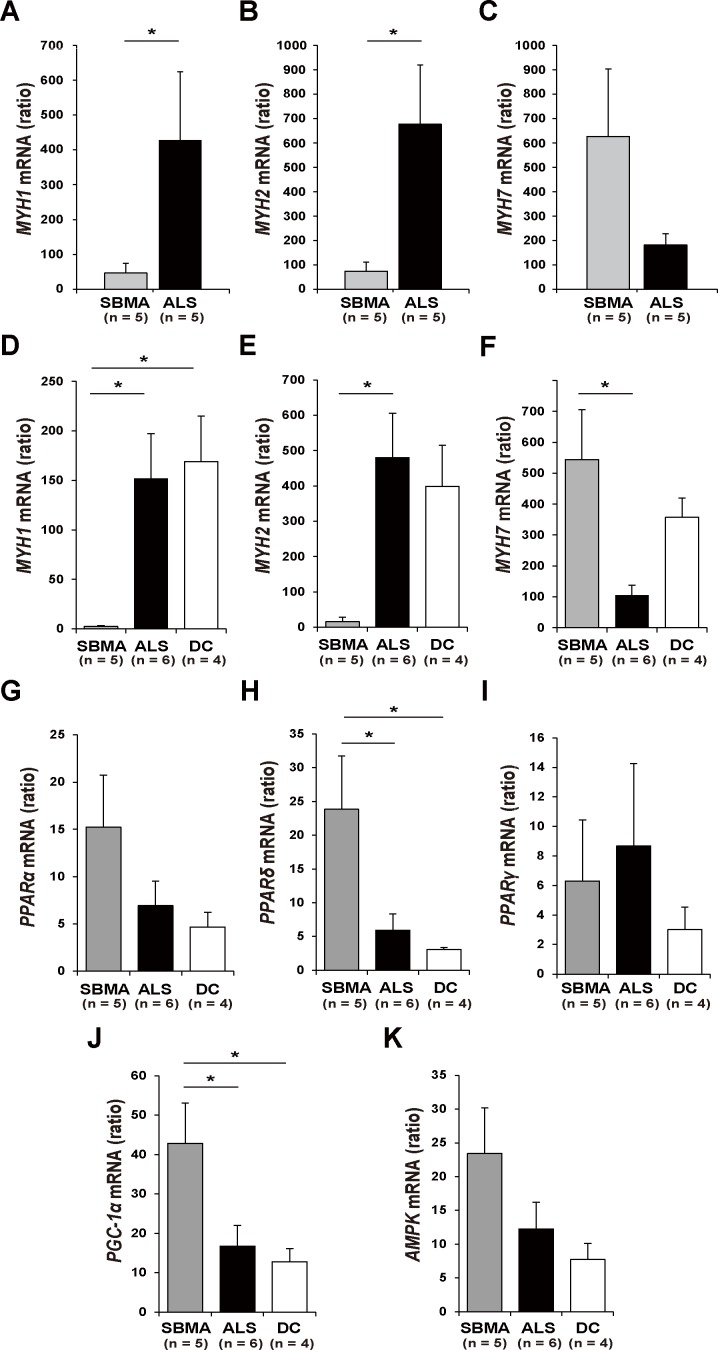
Expression levels of myosin heavy chain and AMPK-PGC-1α pathway in the skeletal muscles. The mRNA expression levels of *MYH1* (encoding MHC type IIx), *MYH2* (encoding MHC type IIa), and *MYH7* (encoding MHC type I) normalized to β2-microglobulin levels in the intercostal muscles of SBMA (*n* = 5) and ALS (*n* = 5) subjects (A–C). The expression levels of *MYH1* (A) and *MYH2* (B) were significantly decreased in subjects with SBMA compared with subjects with ALS. The mRNA expression levels of *MYH1*, *MYH2*, and *MYH7* normalized to β2-microglobulin levels in the iliopsoas muscles of SBMA (*n* = 5), ALS (*n* = 6), and DC (*n* = 4) subjects (D–F). The mRNA levels of *MYH1* and *MYH2* in the iliopsoas muscle were significantly decreased in subjects with SBMA compared with ALS subjects, as observed in the intercostal muscles (D, E). The expression levels of *MYH7* were significantly higher in subjects with SBMA than in subjects with ALS (F). Expression levels of the genes known to regulate muscle fiber type switching in SBMA (*n* = 5), ALS (*n* = 6), and DC (*n* = 4) samples. The mRNA levels of *PGC-1α* and *PPAR-δ*, which regulate the oxidative fiber type profile, were significantly increased in SBMA compared with ALS and DC. The Mann-Whitney U test was performed to assess significant differences for each target gene between SBMA and ALS. ANOVA with Tukey’s post-hoc test was performed to compare the significance of differences in each target gene among SBMA, ALS, and DC. **p < 0.01. *p < 0.05. Data are presented as the mean ± SE. SBMA, spinal and bulbar muscular atrophy; ALS, amyotrophic lateral sclerosis; DC, disease control; *PGC-1α*, proliferator-activated receptor gamma coactivator 1-alpha; *PPAR*, peroxisome proliferator-activated receptor.

To verify the immunofluorescence findings, we performed qRT-PCR analysis on intercostal and iliopsoas muscle specimens. In the intercostal muscles, which generate expiratory flow, the mRNA expression levels of *MYH1* and *MYH2*, encoding MHC type IIx and IIa, respectively, but not *MYH7*, were significantly decreased in samples from SBMA subjects compared with ALS subjects, strengthening the theory that fast muscle power is predominantly affected in SBMA (Fig 5A–5C). A corresponding reduction in *MYH1* and *MYH2* mRNAs was also observed in the iliopsoas muscles (Fig 5D and 5E). By contrast, the expression levels of *MYH7*, which encodes MHC type I, in the iliopsoas muscles were higher in samples from the SBMA subjects than in samples from the ALS subjects (Fig 5F). Furthermore, we compared the expression of putative regulators of muscle fiber switching among the groups (Fig 5G–5K). Results demonstrated that the mRNA levels of *PGC-1α* and *PPARδ*, which are known to regulate oxidative fiber type profile [21,22], were substantially up-regulated in SBMA samples compared with ALS and disease-free control samples (Fig 5H and 5J). Although not significant, *AMPK* mRNA levels tended to be increased in subjects with SBMA (Fig 5K).

## Discussion

In the present study, we demonstrated that %PEF and %FVC were substantially decreased in SBMA compared with controls, although there was no significant difference in FEV_1.0_ between the groups. Both %PEF and %FVC correlated with functional disease scores, particularly truncal subscores, and were linked to decreases in disease progression in subjects with SBMA. PEF is the maximum expiratory flow per minute and can be used to measure how fast a subject can breathe out, providing a reliable global measure of voluntary cough against the risk of aspiration pneumonia [23]. PEF is based on abdominal and intercostal muscle strength as well as the elastic recoil of the lung and chest wall. During forced expiration, such as peak flow, the abdominal muscles are activated to increase intra-abdominal pressure [24]. Furthermore, lower PEF values are associated with increased mortality from respiratory causes [25]. %PEF decreases during the course of neuromuscular disease [26] as well as during spinal cord injuries, in which a higher cord level lesion is associated with a greater decrease in %PEF [27]. As SBMA is a rare disease, clinical trials have to be done with a limited sample size. Therefore, identification of sensitive biomarkers for detecting benefits of tested therapies is urgently needed for SBMA [28]. Our study revealed that %PEF chiefly reflects truncal muscle strength in subjects with SBMA and is a reliable respiratory marker for disease severity and progression.

When comparing SBMA and ALS subjects, only %PEF was significantly decreased in SBMA. Unlike other parameters of pulmonary function tests, PEF is generated by explosive muscle power in an effort-dependent manner. We further revealed that, in addition to %PEF, tongue pressure and grip power, which also reflect explosive muscle strength, are also specifically decreased in subjects with SBMA compared with those with ALS. By contrast, the mQMG score, which is an index of muscle endurance, was similar between the two groups. Taken together, these findings suggest a reduction in explosive muscle strength in subjects with SBMA, consistent with the preferential loss of fast-twitch muscle fibers in these subjects. Muscular function is chiefly dependent on the specific characteristics of various muscle fiber types, and immunohistochemistry of the MHC isoforms in skeletal muscles reveals four fiber types (i.e., I, IIa, IIx, and IIb) in rodents and most other mammalian species. However, only type I, IIa, and IIx fibers are present in most human muscles [29]. These fibers differ from one another in oxidative/glycolytic metabolism: type I fibers are more oxidative and regulate endurance muscle strength; type IIx fibers are more glycolytic and regulate explosive muscle strength; type IIa fibers exhibit characteristics of both type I and IIx fibers [30]. Glycolytic fast-twitch fibers are preferentially vulnerable in a transgenic mouse model of SBMA and in humans with the disorder [31–33]. Results from the present study indicate that the decrease in explosive muscle strength in subjects with SBMA is strongly associated with a reduction in the number of fast-twitch muscle fibers.

In the present study, the muscles of SBMA subjects demonstrated glycolytic-to-oxidative switching in association with an up-regulation of PGC-1α and PPARδ. Fiber type switching is induced in adult skeletal muscle by changes in nerve activity or loading. Glycolytic-to-oxidative switching can be induced by tonic low-frequency electrical stimulation [30]. The AMPK-PGC-1α pathway alters the fiber type profile toward oxidative metabolism by regulating *MYH* gene expression. For instance, overexpressing AMPK and PGC-1α in mice has been shown to increase mitochondrial content and the levels of oxidative enzymes in fast muscle fibers, increasing muscle resistance against fatigue [21,34]. Similarly, PPARδ signaling induces a more oxidative fiber type profile in mice, with an increased amount of mitochondrial DNA, up-regulation of slow contractile protein genes, and an increased resistance to fatigue [22]. PGC-1α was reportedly increased in the muscles of a knock-in mouse model of SBMA, consistent with our findings in humans [35]. Given that AR inhibits the AMPK-PGC-1α pathway [36], the loss of AR function may underlie the up-regulation of PGC-1α in SBMA. In fact, subjects with SBMA often exhibit certain symptoms of androgen insensitivity syndrome, such as gynecomastia and reduced fertility, which have been attributed to loss of AR function [37]. Females possess a greater number of slow-twitch fibers than males, which is the molecular basis for gender differences in response to fatigue or muscle tetanus. These gender differences further support our view that disease-specific alterations of fiber type occur in the skeletal muscles of subjects with SBMA.

A possible alternative explanation for the preferential deficiency of fast-twitch fibers in subjects with SBMA is the adaptation of surviving muscles to endurance activities during the slow progression of the disease. This view is supported by the observation that chronic inactivation of muscles leads to selective atrophy of fast-twitch fibers [38]. A similar loss of fast-twitch fibers was also documented in spinal muscular atrophy, which is another neuromuscular disorder affecting both spinal motor neurons and skeletal muscle [39]. Future studies should directly address the mechanisms underlying disease-specific alterations of fiber type in SBMA, and they should focus on identifying pharmacological or non-pharmacological interventions to reverse these alterations.

In summary, we found that subjects with SBMA exhibited decreased %PEF, which appears to reflect the preferential involvement of fast-twitch fibers in this disease. Given that the leading causes of death in subjects with SBMA are pneumonia and respiratory failure [7], particular attention should be paid to %PEF decline during the clinical management of SBMA.

## Supporting Information

S1 Fig Relationships between %PEF and SBMAFRS scores in subjects with SBMA. Relationships between %PEF and the total score (A) and subscores (B–F) of SBMAFRS in SBMA. %PEF in subjects with SBMA were correlated well with the total scores (A) and trunk (D) and lower limb (E) subscores of SBMAFRS. %PEF, predicted values of peak expiratory flow; SBMAFRS, Spinal and Bulbar Muscular Atrophy Functional Rating Scale; SBMA, spinal and bulbar muscular atrophy.
(PDF)

S2 Fig Relationships between %PEF and ALSFRS-R scores in subjects with SBMA. Relationships between %PEF and the total score (A) and subscores (B–F) of ALSFRS-R in SBMA. %PEF values in subjects with SBMA correlated well with the total score (A) and the trunk (D) and lower limb (E) subscores of ALSFRS-R. %PEF, predicted values of peak expiratory flow; ALSFRS-R, the revised Amyotrophic Lateral Sclerosis Functional Rating Scale; SBMAFRS, Spinal and Bulbar Muscular Atrophy Functional Rating Scale.
(PDF)

S1 Methods (DOCX)
