# Supplementary figures and images for: Decreased Peak Expiratory Flow Associated with Muscle Fiber-Type Switching in Spinal and Bulbar Muscular Atrophy
Source: PLoS One. 2016 Dec 22;11(12):e0168846. doi: 10.1371/journal.pone.0168846 (PMC5179045; doi:10.1371/journal.pone.0168846)

S1 Fig

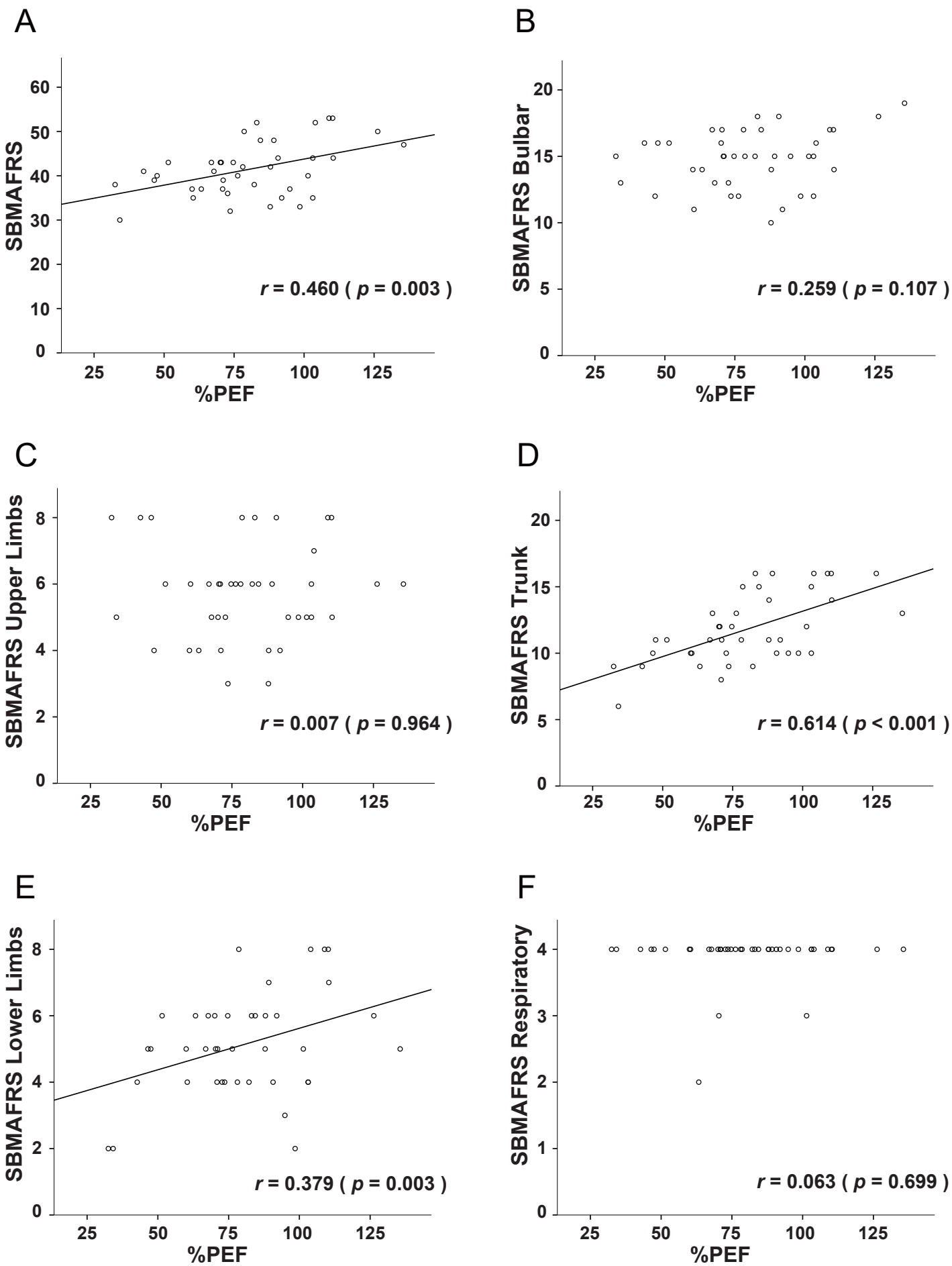

Supplement: S1 Fig — Relationships between %PEF and the total score (A) and subscores (B–F) of SBMAFRS in SBMA. %PEF in subjects with SBMA were correlated well with the total scores (A) and trunk (D) and lower limb (E) subscores of SBMAFRS. %PEF, predicted values of peak expiratory flow; SBMAFRS, Spinal and Bulbar Muscular Atrophy Functional Rating Scale; SBMA, spinal and bulbar muscular atrophy. (PDF) [file pone.0168846.s001.pdf]

S2 Fig

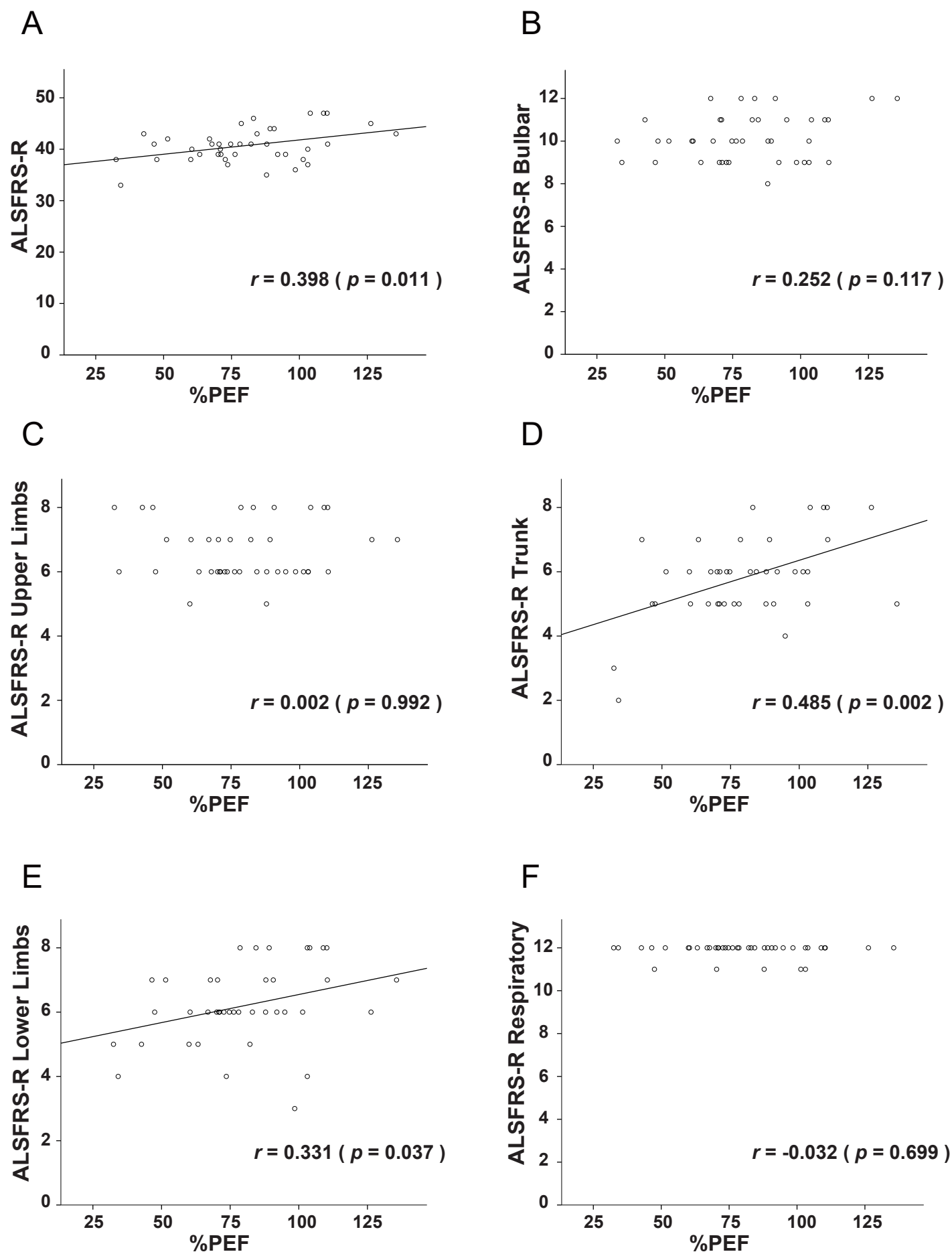

Supplement: S2 Fig — Relationships between %PEF and the total score (A) and subscores (B–F) of ALSFRS-R in SBMA. %PEF values in subjects with SBMA correlated well with the total score (A) and the trunk (D) and lower limb (E) subscores of ALSFRS-R. %PEF, predicted values of peak expiratory flow; ALSFRS-R, the revised Amyotrophic Lateral Sclerosis Functional Rating Scale; SBMAFRS, Spinal and Bulbar Muscular Atrophy Functional Rating Scale. (PDF) [file pone.0168846.s002.pdf]
