# Supplementary material for: Decreased Peak Expiratory Flow Associated with Muscle Fiber-Type Switching in Spinal and Bulbar Muscular Atrophy
Source: PLoS One. 2016 Dec 22;11(12):e0168846. doi: 10.1371/journal.pone.0168846 (PMC5179045; doi:10.1371/journal.pone.0168846)
Supplement: S1 Methods — (DOCX) [file pone.0168846.s003.docx]

# **Supporting Information**

# **S1 Methods**

# **Motor functions**

The ALSFRS-R is a validated, questionnaire-based scale that measures physical function in patients with ALS. All items on the ALSFRS-R are also generally applicable to patients with SBMA [1]. The SBMAFRS is another validated functional rating scale which has been utilized to assess the functional status of patients with SBMA [2–4]. The QMG score is an objective measurement used to detect the fatigue of endurance muscle power [5]. We used a part of the QMG score that measures the muscle endurance of outstretched extremities and the lifted head as the mQMG; the best possible score is 0 and the worst possible score is 15. Grip power was measured using an electronic hand dynamometer (TTM digital dynamometer 110kgYD; Tsutsumi Seisakujyo, Kamagaya, Japan). For measurement, the subjects were instructed to keep their elbows at an angle of 90°, their forearms at a neutral rotation, and their wrists not flexed or pronated. We recorded the maximal power of the dominant hand. Tongue pressure was evaluated using a digital tongue pressure measurement device (JMS Co., Ltd., Hiroshima, Japan). We asked the participants to compress the balloon of a disposable intraoral pressure probe upward onto their palates for 7 s using the maximum voluntary effort of the tongue. We recorded tongue pressure (kPa) 3 times at 1 min intervals, and adopted the maximum pressure recorded as the maximal tongue pressure for each time. We used maximal tongue pressure for our analyses [6]. We assessed all measures of motor function, except for the mQMG, in the healthy controls.

# **Quantitative RT-PCR**

Individual 50 mg frozen samples of muscle were homogenized, and total RNA was extracted using TRIzol (Invitrogen, CA, USA) as described previously [7]. The extracted RNA was then reverse-transcribed into first strand cDNA using a ReverTra Ace^®^ qPCR RT Kit (TOYOBO, Osaka, Japan). Quantitative RT (qRT)-PCR was performed in a total volume of 20 µL that contained 10 µL of 2× KOD SYBR^®^ qPCR Mix (TOYOBO, Osaka, Japan) and 40 µM of each primer (Sigma-Aldrich, St Louis, MO, USA); the amplified products were detected with the CFX96™ Real-Time System (Bio-Rad Laboratories, Hercules, CA, USA). The reaction conditions were 98°C for 2 min and 40 cycles of 10 s at 98°C, 10 s at 60°C, and 30 s at 68°C. The expression level of the internal control β2-microglobulin was quantified simultaneously. Relative gene expression was determined using the ΔΔCT method. The following primers were used: 5′-GACAACTCCTCTCGCTTTGG-3′ and 5′-GGCATAATCGTATGGGTTGG-3′ for *MYH1*; 5′-GATGGCACAGAAGTTGCTGA-3′ and 5′-CTTCTCGTAGACGGCTTTGG-3′ for *MYH2*; 5′-TGTGTCACCGTCAACCCTTA-3′ and 5′-TGGCTGCAATAACAGCAAAG-3′ for *MYH7*; 5′-CCTGCATGAGTGTGTGCTCT-3′ and 5′-Gcaaagaggctggtcttcac-3′ for *PGC-1α*; 5′-gtttgagggggtaacagcaa-3′ and 5′-gctaactgcagagggtgagg-3′ for *PPARα*; 5′-actgagttcgccaagagcat-3′ and 5′-gcgttgaacttgacagcaaa-3′ for *PPARδ*; 5′-gctgtgcaggagatcacaga-3′ and 5′-gggctccataaagtcaccaa-3′ for *PPARγ*; 5′-ggagccttgatgtggtagga-3′ and 5′-tttcatccagccttccattc-3′ for *AMPK*; and 5′-TTTCATCCATCCGACATTGA-3′ and 5′-CCTCCATGATGCTGCTTACA-3′ for β2-microglobulin*.*

# **References**

1. Banno H, Katsuno M, Suzuki K, Takeuchi Y, Kawashima M, Suga N, et al. Phase 2 trial of leuprorelin in patients with spinal and bulbar muscular atrophy. Ann Neurol. 2009;65:140–150.

2. Hashizume A, Katsuno M, Suzuki K, Banno H, Suga N, Mano T, et al. A functional scale for spinal and bulbar muscular atrophy: Cross-sectional and longitudinal study. Neuromuscul Disord. 2015;25:554–562.

3. Hijikata Y, Katsuno M, Suzuki K, Hashizume A, Araki A, Yamada S, et al. Impaired muscle uptake of creatine in spinal and bulbar muscular atrophy. Ann Clin Transl Neurol. 2016;3:537-546.

4. Querin G, DaRe E, Martinelli I, Bello L, Bertolin C, Pareyson D, et al. Validation of the Italian version of the SBMA Functional Rating Scale as outcome measure. Neurol Sci. 2016;21. [In press]

5. Besinger UA, Toyka KV, Hömberg M, Heininger K, Hohlfeld R, Fateh-Moghadam A. Myasthenia gravis: long-term correlation of binding and bungarotoxin blocking antibodies against acetylcholine receptors with changes in disease severity. Neurology. 1983;33:1316–1321.

6. Mano T, Katsuno M, Banno H, Suzuki K, Suga N, Hashizume A, et al. Tongue pressure as a novel biomarker of spinal and bulbar muscular atrophy. Neurology. 2014;82:255–262.

7. Araki A, Katsuno M, Suzuki K, Banno H, Suga N, Hashizume A, et al. Brugada syndrome in spinal and bulbar muscular atrophy. Neurology. 2014;82:1813–1821.
